# Environmental sustainability in medical school curriculum: a scoping review

**DOI:** 10.1186/s12909-026-09617-6

**Published:** 2026-06-02

**Authors:** Jasmine G. Hughes, Samuel Y. Kim, Divyansh Agarwal, Veronica Phillips, Craig D. McClain

**Affiliations:** 1 Department of Clinical Neurosciences, University of Cambridge, Cambridge, UK; 2 Department of Anesthesiology, Critical Care, and Pain Medicine, Boston Children’s Hospital, Boston, MA USA; 3 Department of Surgery, Massachusetts General Hospital, Boston, MA USA; 4 University of Cambridge Medical Library, University of Cambridge, Cambridge, UK; 5 Program in Global Surgery and Social Change, Harvard Medical School, Boston, MA USA

**Keywords:** sustainability, medical education, environmental impact

## Abstract

**Background:**

In this scoping review, we aimed to explore the structure, topics, and objectives presented within environmental sustainability curricula in medical education and assess the impact and quality of environmental sustainability curricula in the medical schools.

**Methods:**

We conducted searches in Medline, Embase, Web of Science, Cinahl, and Global Index Medicus from inception to October 2025. Full-text, peer-reviewed articles were evaluated based on inclusion criteria. Data extracted from included articles included author, year, location, curriculum design, objectives, topics, outcome measures, and main findings.

**Results:**

The searches resulted in 3357 original articles. A total of five publications met the inclusion criteria and were dated from 2021 to 2025. The structure of the environmental sustainability curriculum in medical education reported in the studies included a one-hour interactive session, a 10-week traditional seminar elective, and a longitudinal integrated societal theme. Studies reported a significant improvement in medical students’ self-perceived competence and knowledge in climate-related health issues.

**Conclusion:**

Although the included studies highlighted a few different structures of incorporating environmental sustainability in medical education, there still remains a gap in the current literature on how best to implement and objectively assess the impact. Therefore, more research on environmental sustainability curriculum in medical education is needed.

**Supplementary Information:**

The online version contains supplementary material available at 10.1186/s12909-026-09617-6.

## Introduction

Climate change is the biggest global health threat of the 21st century. [1] The United States (US) healthcare system accounts for 25% of the global health sector’s greenhouse gas emissions (GHGe) and 8.5–10% of the US’s total GHGe. [2–5] Climate change influences how care is delivered, increases medical costs, impacts mental health, and intensifies the risk of natural disasters. [6–11] This inevitably will disproportionately affect those of lower socioeconomic status. [12, 13] In the US, many medical schools have adopted an ethical oath with an emphasis on non-maleficence, modeling the Hippocratic Oath. [14, 15] With this declaration, physicians have an obligation to not only protect patients from harm but also to consider how to prevent harm locally and globally.

Several have already advocated for the implementation of climate education into the medical curricula. In 2018, Wellbery et al. worked alongside the Association of American Medical Colleges (AAMC) to query their curriculum inventory, which aggregates institution-reported curricular activity, but the search indicated that schools do not report any explicit inclusion of climate change education in their curriculum. [16] In 2019, medical students at the University of San Francisco standardized a Planetary Health Report Card for the evaluation of medical schools to help inspire change at the institutional level. [17] In 2024, Cois et al. examined curricula in graduate medical education (GME) programs. [18] Finally, Hampshire et al. conducted a survey of 722 medical students from 24 medical schools. Of the 600 respondents, approximately 84% of students believed that climate change and health effects should be included, yet only 13% believed that their school provided adequate education. [19].

Given the recent rollback of US federal regulations and agencies supporting climate initiatives, including the US withdrawal from the Paris Climate agreement and the United Nations Framework on Convention on Climate Change (UNFCCC), it is imperative to incorporate the realities of climate change and its impact in medicine. [20, 21] In order to better equip future physicians, climate education and environmental sustainability must be incorporated into the medical school curricula. Therefore, in this scoping review, we aimed to survey the existing literature on how climate change content is being currently integrated into medical school curricula within the US and highlight the feasibility of educational interventions based on institutional needs.

## Methods

### Search strategy and information sources

The databases Medline (via Ovid), Embase (via Ovid), Web of Science (Core Collection), Cinahl (via Ebscohost) and Global Index Medicus were searched from inception to October 2025 by VP. The search strategy was peer-reviewed by two librarian colleagues of VP using the Peer Review of Electronic Search Strategies (PRESS) checklist, [22] and evaluated against the PRISMA-S guidelines. [23] Databases were searched by VP separately, rather than multiple databases being searched on the same platform. The search syntax was adapted for each database, and to account for variation between thesaurus terms/controlled vocabulary across each database. Results were imported to Endnote 21 by VP for deduplication, using the method outlined by Bramer et al. [24]Dates in which the searches were run, and the full search strategies used in each database, are documented in the supplemental materials.

### Inclusion and exclusion criteria

Studies were considered eligible if they met the following inclusion criteria: full-text, published, peer-reviewed empirical studies with a focus on integrating environmental sustainability into medical school curriculum with students. Studies conducted outside of the US and non-English publications were excluded. Editorials, book chapters, letters to the editor, conference abstracts, and proceedings were also excluded.

### Study selection and data extraction

Titles and abstracts were screened on the Covidence (Covidence systematic review software, Veritas Health Innovation) for subsequent full-text review for those included after this screening. A template for data extraction was used for selected studies, which included the following information: author, year, country, institution, curriculum topics, and reported outcomes. Data extraction was completed by JH and SK. After consensus was reached, the extracted data sheets were assessed by JH.

### Narrative synthesis

A narrative synthesis was conducted in accordance with the objectives of the scoping review. The synthesis consists of two parts: (i) exploring the structure, topics and objectives of environmental sustainability in medical education curricula used in the studies, and (ii) assessing the impact of integrating environmental sustainability into the medical school curricula. A minimum of two studies was required for the narrative synthesis.

### Quality assessment

The validity and methodology quality of the included articles were assessed using the Medical Education Research Study Quality Instrument (MERSQI). [25] Each article was scored on a total range of 5 to 18 based on the following MERSQI domains: study design, sampling, type of data, evidence of validity, data analysis, and outcomes. Two reviewers graded each article independently, and a third reviewer was available to discuss and resolve any conflicts.

## Results

### Search results

**Fig. 1:**
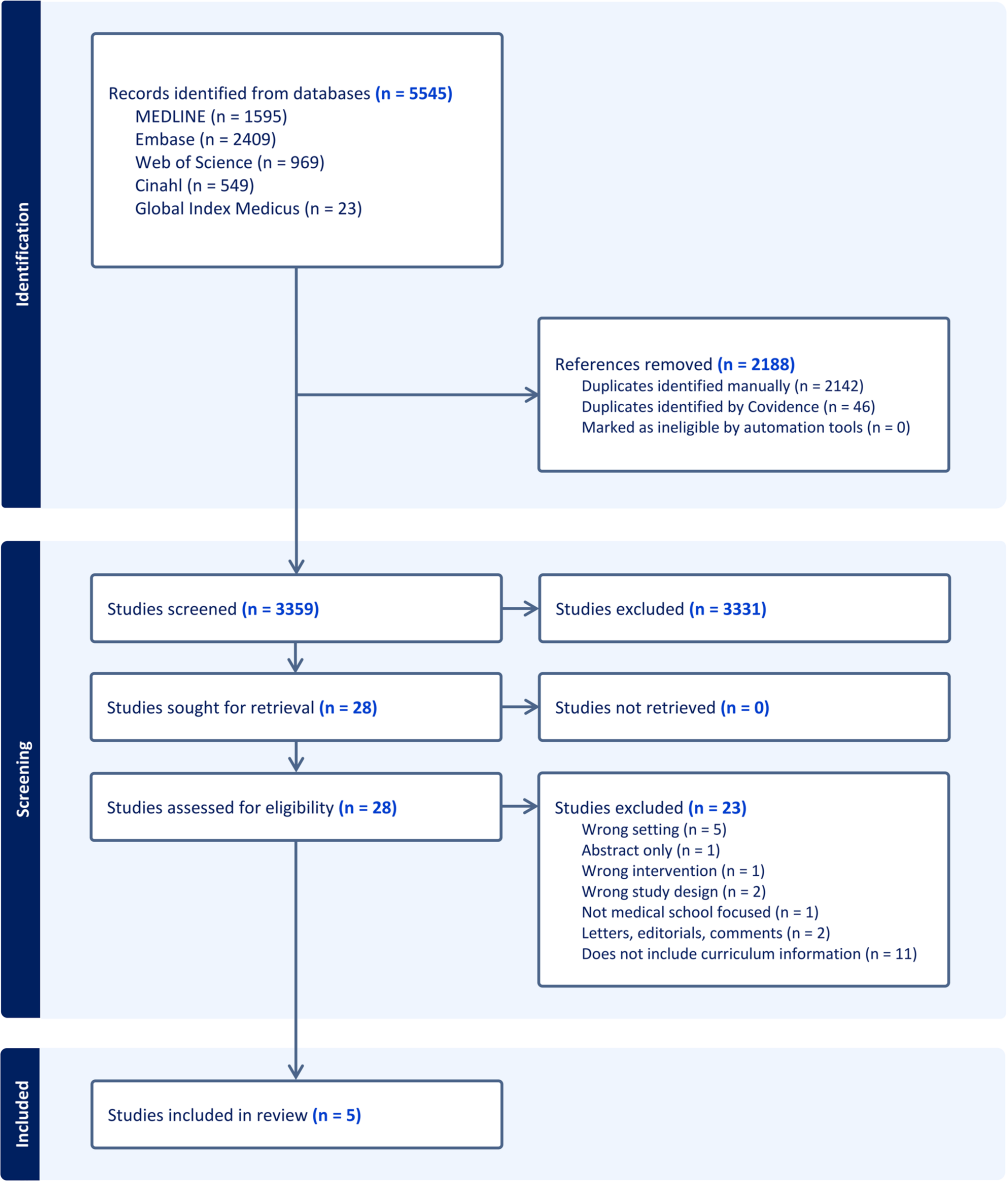
PRISMA Flow Diagram

The systematic search across all databases resulted in 5545 records. After the removal of duplicates in EndNote, a total of 3403 records were imported into Covidence. Further duplicates were identified and removed in Covidence, which resulted in 3357 original articles. Of these studies, 3331 were excluded during the title and abstract screening. Of the remaining 28 studies, 23 did not meet the inclusion criteria and were removed. This resulted in five articles being included in our review. The screening and selection processes are shown in the PRISMA flowchart (Fig. 1).

### Design and characteristics of included studies

The five included studies were published between 2021 and 2025, as noted in Table 1. A total of 394 medical students were participants in the studies, with two studies including only first-year medical students, one study with only second-year medical students, and one study consisting only of third-year medical students. For three studies, the course was required for medical students, and in two studies, the course was an optional elective.

**Table 1:** Characteristics of Included Studies

Author	Year	Study Location	Sample Size	Curriulum Design	Objectives of Educational Intervention	Topics in Educational Intervention	Outcome Measures	Main Findings
Baker et al. [26]	2025	Harvard Medical School, Boston, MA, United States of America	134	Longitudinal, integrated Climate Change, Environment, and Health (CCEH) societal theme	1. Define the pathophysiological mechanisms by which climate change, air pollution and ecological degradation impact human health. 2. Apply knowledge of climate impacts on human health to the clinical care of patients including prevention, diagnosis, and risk reduction counseling. 3. Analyze the historical and structural causes of climate change, air pollution and ecological degradation, and describe the ways in which it creates/exacerbates health inequity. 4. Describe the ways in which the health system contributes to climate change and how healthcare delivery is vulnerable to climate-related events. 5. Explore roles health professionals and institutions can play in climate solutions.	Advocacy and activism through a climate lens; Land use change and malaria; Exposure history and heat risk screening tool; Hurricanes and waterborne disease; Environmental and global climate justice; Air pollution, asthma, and cardiovascular health; Communicating polarizing science topics (like climate change, as physicians); Heat exposure and kidney disease, dialysis access; Allergic rhinitis and climate change; Climate change and mental health; Healthcare sustainability and waste, heat stress and geriatrics, cardiovascular and respiratory consequences of air pollution	Pre- and post competency-based survey and course content-specific survey	Comparing pre-to post- course responses, there was a statistically significant improvement in both the competency-based and course content-specific surveys. The climate change, environment and health curricular theme improved students’ self perceived competence and knowledge in climate related health issues.
Costin et al. [27]	2024	Wright StateUniversity Boonshoft School of Medicine; Dayton, OH, United States of America	70	One hour interactive educational session on climate change and mental health	1. List psychiatric conditions and other mental health impacts that emerge from and/or are affected by the climate crisis. 2. Describe methods of climate communication in the clinical setting. 3. Compare roles that health professionals should perform infacilitating resilience and pro-environmental behaviors. 4. Describe available resources to facilitate such public health and mental health activities.	An overview of mental health impacts from climate change and its drivers (i.e., drought, sea level rise, air pollution, wildfires, severe storms, and extreme heat); Psychological effects from and responses to climate change; Actionable items enabling health care providers to address climate change along with a list of currently available resources.	Pre- and postsession content survey	There was a statistically significant improvement in average score on the content survey post-session (2.7 pre-session to 3.9 post-session). Qualitative analysis identified knowledge gained about the mental health impacts of climate change as the most important aspect of the session to students.
Dalapati et al. [28]	2024	Duke University School of Medicine, Durham, NC, United States of America	118	The Climate Change, Health, and Equity thread embedded into the required 21-week Foundations of Patient Care 2 (FPC2) course	1. Understand the basic concepts of the planetary health framework. 2. Identify effects of climate change on physical and mental health. 3.Describe climate change as a social driver of health in the local community. 4. Recognize examples of how climate policy disproportionately affects the health and equity of communities of color and of lower socioeconomic status. 5. Recognize the importance of and learn from the lived experiences of communities disproportionately affected by climate change. 6. Identify opportunities to advocate and partner with patients, community members, and policymakers to promote health equity and climate resilience. 7. Establish personal and professional accountability for continued self-directed learning.	Planetary Health and Disaster Justice; Health and Climate Change; Mental Health and Climate Change; Climate Change, Policy, and Public Health; Climate Change: An Ecological and Health Equity Crisis; Environmental Justice and Advocacy by Students; Beyond PubMed: Databases, Sources and Appraisal	Critical reflection essay	Thematic analysis showed students were keen at identifying new areas of medical knowledge and connecting concepts to individual experiences, institutional practices, and public health and policy. Most, 90% of students expressed negative feelings like frustration and fear but also positive sentiments of solidarity and hope regarding climate change and effects on health. Approximately 80% of students expressed actionable items at every level including continuing self-directed learning and conversing with patients, minimizing health-care waste, and advocating for climate-friendly policies.
Navarrete-Welton et al. [30]	2022	Warren Alpert Medical School of Brown University, Providence, RI, United States of America	Not specified	A medical student-driven planetary health curriculum	1. Be able to effectively communicate with patients about the health impacts of climate change, strategies to prevent those risks, and the concept of health co-benefits of action. 2. Demonstrate effective communication with stakeholders about climate and health topics and work collaboratively and across disciplines on climate and health issues. 3. Learn to take an environmental history. 4. Define climate drivers (both natural and human-caused), weather, climate change, and climate variability. 5. Identify the health impacts of climate change and other human-driven disruptions to our natural environment. 6. Apply knowledge of the connection between habitat and biodiversity loss and infectious diseases. 7. Apply knowledge of climate and health to clinical care of patients. 8. Understand how to identify, prevent, and treat commonly seen health related impacts of climate change, i.e., heat stroke, asthma exacerbations, acute kidney injuries, adverse birth outcomes. 9. Recognize the disproportionate impact of climate change on communities of color and explain pre- existing and future health disparities rooted in environmental racism, especially in Rhode Island. 10. Apply climate and health knowledge to improve decisions about public health services, and adapt and improve population health. 11. Describe ways medical students and health professionals can engage in institutional, community, and political advocacy around planetary health. 12. Describe ways that healthcare professionals and facilities can prepare for and respond to climate-related health risks. 13. Appreciate the role of the healthcare industry in contributing to climate change and identify ways healthcare providers can reduce/mitigate waste and carbon emission.	Pathophysiology of major afflictions of the CNS and PNS; Pathophysiology of common neurologic and psychiatric disorders; Epidemiology and risk factors of infectious diseases; Risk factors and pathogenesis of common cardiovascular disorders; Risk factors and pathogenesis of common pulmonary disorders; Risk factors and pathogenesis of common pulmonary disorders; Risk factors andpathogenesis of common reproductive disorders; Risk factors and pathogenesis of common reproductive disorders	None	No formal assessments of the impact were conducted. However, the PHCIC plans to repeat the PH education needs assessment and use structured surveys to assess the effect of the curriculum changes that will be implemented in the future.
Gomez et al. [29]	2021	Stanford University School of Medicine, Stanford, CA, 94305, United States of America	72	10-week traditional, graduate level, seminar style elective	1. Explain links between the environmental effects of climate change and human health. 2. Describe how climate change shapes healthcare delivery, from individuals to systems. 3. Form and defend a position on the effects of climate change on human health. 4. Apply principles of sustainability and environmental health counselling to clinical practice. 5. List three behavioral changes in one's personal or professional life that may potentially counteract the environmental effects of climate change.	Climate Change and Health Overview; Climate Change and Mental Health; Climate Change and Infectious Disease; Climate Change and Pediatric Health; Climate Change and Women’s Health; Environmental Justice; Climate-Related Disasters and Disaster Response; Climate-Smart Health Care; Food Waste, Security, and Nutrition; From Clinic To Community- Patient and Community Panel	Pre and-post intervention survey	There was a significant increase found in students' beliefs about the need for climate change education and related physician responsibilities and their intentions to change personal behaviors and apply new knowledge in clinical practice.

### Narrative synthesis

#### Structure, topics and objectives of environmental sustainability educational intervention

The studies reported a range of structures for an educational intervention, including a one-hour interactive session, a 10-week traditional seminar elective, a climate change, health, and equity thread embedded into the required 21-week Foundations of Patient Care 2 (FPC2) course, and a longitudinal, integrated climate change, environment, and health societal theme. [26–30] A variety of topics were mentioned in the included studies, which included advocacy and activism through a climate lens, climate-smart health care, climate change: an ecological and health equity crisis, healthcare sustainability and waste, climate change and infectious disease, climate change and pediatric health, climate change and women’s health, and actionable items enabling health care providers to address climate change. [26–30].

The Climate Change and Mental Health session’s educational objectives included to list psychiatric conditions that emerged from or were affected by the climate crisis, comparison of roles in which health professions should perform to facilitate resilience and pro-environmental behaviors and describe methods of climate communication in a clinical setting. [27].

The Climate Change, Health, and Equity thread’s objectives consists of students understanding the basic concepts of planetary health framework, identifying effects of climate change on physical and mental health, describing climate change as a social driver of health in the local community, recognizing the importance and learning from lived experiences of communities disproportionately affected by climate change, and identifying opportunities and partnerships to promote health equity and climate resistance. [28].

The Impact of Climate Change on Human Health course was designed based on Kern’s Six Steps of Curriculum Development and consists of the following five learning objectives: explaining links between environmental effects of climate change and human health, describing how climate change shapes healthcare delivery, forming and defending a position on the effects of climate change on human health, applying principles of sustainability and environmental health counselling to clinical practice, and listing three behavioral changes in one’s personal or professional life that may counteract environmental effects of climate change. [29]

The integrated Climate Change, Environment, and Health (CCEH) societal theme objectives were based on a competency framework which includes the following five core competencies: defining the pathophysiological mechanism by which climate change, air pollution and ecological degradation impact human health, applying knowledge of climate impacts on human health to the clinical care of patients, analyzing the historical and structural causes of climate change and describing the ways in which it creates health inequity, describing the ways in which the health system contributes to climate change, and explore the roles health professionals can pay in climate solutions. [26].

The medical student-driven planetary health curriculum objectives were developed based on the integration of the Global Consortium on Climate and Health Education (GCCHE) Climate and Health Key Competencies for Health Professions Students and the existing framework of the Warren Alpert Medical School’s Nine Abilities. The planetary health curriculum core competencies includes the following: identifying the health impacts of climate change, describing the ways medical students and health professionals can engage in political advocacy centers on planetary health, learn to take an environmental history, applying knowledge of climate and health to clinical care, be able to effectively communicate with patients about the health impacts of climate change, and understand how to identify, prevent and treat commonly seen health related impacts of climate change. [30].

#### Outcomes of environmental sustainability intervention

Studies reported an overall improvement in students’ self-perceived competence and knowledge in climate-related health issues, a significant increase in participants’ intentions to change personal behaviors, the application of new knowledge in future clinical practice, and beliefs about the need for climate change education and related physician responsibilities. [26–30].

A variety of measurements were used to assess outcomes among studies, except in Navarrete-Welton et al. study as a formal outcome measurement was not utilized evaluate the medical student-driven planetary health curriculum. [30] Measurement outcomes used to evaluate and assess the efficacy of the Climate Change and Mental Health session meeting its objectives within the Costin et al. study included a large-group discussion among participants and pre- and post-session surveys. [27] The pre- and post-session surveys consists of the same four questions based on the study’s educational objectives and short answer questions (only in the post-session survey) on what content students found to be the most important learned and suggestions for improvement. [27] Gomez and colleagues, employed a pre-and post-intervention survey based on the categories of ‘Beliefs and Attitudes’ and ‘Knowledge and Application’, to evaluate participants learning from the Impact of Climate Change on Human Health course and their intention to implement behaviors in clinical practice. [29] A similar approach was also seen in Baker et al., as two types of surveys were administered: competency-based survey and content-specific pre-and-post surveys to determine the impact of the climate change, environmental, and health curriculum. [26] The competency-based survey differ from the content-specific survey as the prior assessed the development of the core competencies, while the later assessed the participants learning of specific course topics. [26] In comparison, a qualitative method for evaluation was used in Dalapati et al. study, in which students completed a critical reflection essay at the conclusion of the Climate Change, Health, and Equity thread. [28].

#### Quality assessment

The MERSQI scores of the five included studies ranged from 7 to 10, with an average of 9.1 (SD = 0.74) out of a possible 18 points (Table 2). While none of the included studies consists of a two-group comparison, four studies included pre- and post-tests. The sampling was completed at only one institution for all of the studies. Only two studies included data analysis beyond descriptive statistics. One study did not use any outcome measure to determine the impact of educational intervention, while the remaining four studies only included outcome measures for satisfaction, attitudes, perceptions, opinions, and general facts.

**Table 2:** Medical Education Research Study Quality Instrument (MERSQI)

MERSQI Item	Possible Item Score	Baker et al. 2025 [26]	Costin et al. 2024 [27]	Dalapati et al. 2024 [29]	Navarrete-Welton et al. 2022 [30]	Gomez et al. 2021 [29]
1. Study Design						
Single-group cross sectional or single-group post test only	1			1	1	
single-group pre- and post-tests	1.5	1.5	1.5			1.5
Non-randomized, 2 groups	2					
Randomized control trial	3					
2. Sampling						
Institutions						
- Single institution	0.5	0.5	0.5	0.5	0.5	0.5
- Two institutions	1					
- 2 or more institutions	1.5					
Response Rate						
- <50% or not reported	0.5	0.5			0.5	
- 50-74%	1		1			1
- >74%	1.5			1.5		
3. Type of Data						
Assessment by study subject	1	1	1	1	1	1
Objective measurement	3					
4. Evaluation of Assessment Tool						
Content						
- Not reported	0					
- Reported	1	1	1	1	1	1
Internal Structure						
- Not reported	0					
- Reported	1	1	1	1	1	1
Relationships to Other Variables						
- Not reported	0	0	0	0	0	0
- Reported	1					
5. Data Analysis						
Complexity of Analysis						
- Descriptive Only	1		1	1	1	
- Beyond descriptive	2	2				2
Appropriateness of Analysis						
- Inappropriate	0					
-Appropriate	1	1	1	1	1	1
6. Outcomes						
Satisfaction, attitudes, perceptions, opinions, general facts	1	1	1	1	1	1
Knowledge, skills	1.5					
Behaviors	2					
Patient/health care outcomes	3					
Total	18	9.5	9	9	8	10

## Discussion

The multifaceted ways in which climate change affects human health compel a reconceptualization of conventional distinctions between population health and individual clinical care. Whether it is the seemingly benign impact on allergenic burden, exacerbation of pulmonary irritants, or how carbon footprint affects surgical care, changing environmental processes ultimately manifests as clinical symptoms at the level of individual patients. [31] It is imperative to introduce this reality in medical education, ideally as longitudinal inclusion across the continuum of medical training, as well as into continuing medical education and faculty development initiatives. The goal of this work was to systematically survey existing literature on how climate change content is being integrated into medical school curricula within the US, as well as highlight any educational interventions that may have resulted from such initiatives.

Our results highlight that integration of climate change in medical education can range from brief interactive sessions to longitudinal, fully integrated modules in the curricula. It is important to note that in many medical schools, the curriculum might provide natural entry points for incorporating climate change education through an expansion of the contextual framework in which existing clinical concepts are placed. For instance, a cardiology module might be a natural segue to introducing the harmful effects of air quality degradation on cardiovascular health. Across studies, interventions were associated with increased self-reported knowledge and competence, greater intentions to modify personal behaviors, anticipated integration of climate–health principles into future clinical practice, and strengthened recognition of the importance of climate change education and physician responsibility.

Altogether, integrating climate change into medical education is critical to preparing future physicians to effectively respond to one of the most far-reaching determinants of human health in the 21st century. The five articles included in this scoping review all substantiate how formal instruction in climate–health relationships equips medical trainees with the competencies required to identify and manage climate-related health risks, provide informed patient counseling, adapt clinical and public health practices, and engage in mitigation and advocacy efforts. Yet, there is a paucity of literature documenting tangible implementations of climate health integration into the US medical education and how such initiatives might affect training in medical schools.

Existing curricular and institutional frameworks for integration of environmental sustainability in medical school curriculum outside of the US can serve as models for future research. In the United Kingdom (UK), the Sustainability in Quality Improvement (SusQI) framework consists of four steps which combines social and environmental sustainability with a quality improvement methodology. [32] The integration of the SusQI framework into medical education at the University of Bristol, has resulted in an increase students’ motivation and engagement for both quality improvement and sustainable health care. [33] The Planetary Health Education (PHE) framework established by the Australian Medical Council, comprised of standards centered on recognition of health impacts of climate change and the pressing need for environmental sustainability into medical practice. [34] Based on the PHE framework, the inclusion of Doctors for the Environment Australia (DEA) educational resources on climate, health and sustainability into the Royal Melbourne Hospital curriculum, led to a significant increase in students’ knowledge and understanding of the potential health impacts of climate change, the environmental impact of healthcare delivery and the health co-benefits of climate action. [35].

The included studies reflect elements of several established pedagogical frameworks that warrant explicit adoption in future research. Competency-based education is evident in studies that structured learning objectives around defined clinical and advocacy competencies, [26, 30] while approaches consistent with reflective and transformative learning are seen in the use of critical reflection essays as an outcome measure. [28] The use of Kern’s Six Steps of Curriculum Development [29] and student-driven curriculum models [30] further reflect experiential and problem-based learning approaches across the included interventions. Future research should deliberately articulate and evaluate these frameworks to move beyond descriptive outcomes toward a more theoretically grounded understanding of how climate health education changes physician thinking and practice.

We surmise that more schools will begin to incorporate climate change into the medical curriculum, and we anticipate that this, in turn, would contribute to the development of resilient and sustainable health care systems.

### Limitations

There are some limitations to note within this scoping review. As part of the eligibility criteria, this scoping review only included peer-reviewed published studies. Literature covering this topic in other types of publications and media may not been discussed. The small size of the included studies can limit the generalizability of the findings. Each study consisted of only one cohort of participants, the current year in medical school for participants varied across the studies, and each study only consisted of descriptive analysis. Therefore, the reported impact and validity of the educational interventions can be biased. In addition, there is bias in representation as the majority of the studies were conducted at private medical schools. As such, the impact of such educational interventions may not be transferable to publicly funded or low-resource medical schools.

## Conclusion

This scoping review resulted in a variety of methods to introduce environmental sustainability in medical school curricula, which ranged from one-hour interactive sessions to a longitudinal, integrated societal theme. However, due to a current sparsity in the literature on how to implement environmental sustainability in medical school curricula and to measure the impact objectively, more research is urgently warranted.

## Supplementary Information

Supplementary Material 1.

## Data Availability

The datasets used and/or analyzed during the current study are provided in supplemental materials.

## Abbreviations

AAMC: Association of American Medical Colleges
CCEH: Climate Change, Environment, and Health
DEA: Doctors for the Environment Australia
FPC2: Foundations of Patient Care 2
GHGe: Greenhouse gas emissions
GCCHE: Global Consortium on Climate and Health Education
GME: Graduate medical education
MERSQI: Medical Education Research Study Quality Instrument
PHE: Planetary Health Education
PRESS: Peer Review of Electronic Search Strategies
PRISMA: Preferred Reporting Items for Systematic Reviews and Meta-Analyses
PRISMA-S: Preferred Reporting Items for Systematic reviews and Meta-Analyses literature search extension
SusQI: Sustainability in Quality Improvement
UK: United Kingdom
UNFCCC: United Nations Framework on Convention on Climate Change
US: United States
