# Supplementary material for: Environmental sustainability in medical school curriculum: a scoping review
Source: BMC Med Educ. 2026 Jun 2;26:1252. doi: 10.1186/s12909-026-09617-6 (PMC13445735; doi:10.1186/s12909-026-09617-6)
Supplement: Supplementary file 1 [file 12909_2026_9617_MOESM1_ESM.docx]

**SUPPLEMENTAL MATERIALS**

Search Strategies

Ovid MEDLINE(R) and Epub Ahead of Print, In-Process, In-Data-Review & Other Non-Indexed Citations, Daily and Versions <1946 to October 03, 2025>

1	((medical or clinical or doctor* or healthcare) adj5 (student* or educat* or school* or universit* or learn*)).ti,ab,kw,kf.	324688

2	curriculum/ or education, premedical/ or exp education, medical/	253370

3	1 or 2	490783

4	exp Climate Change/ or exp Environmental Health/	67588

5	("climate change" or "global warming" or "global heating" or "climate crisis" or "environmental health").ti,ab,kw,kf.	111977

6	4 or 5	143746

7	3 and 6	1595

Embase <1974 to 2025 October 02>

1	curriculum/ or curriculum development/	125310

2	exp medical education/	407132

3	medical student/	104981

4	((medical or clinical or doctor* or healthcare) adj5 (student* or educat* or school* or universit* or learn*)).ti,ab,kw,kf.	458223

5	1 or 2 or 3 or 4	801424

6	exp climate change/	83948

7	exp environmental health/	37055

8	("climate change" or "global warming" or "global heating" or "climate crisis" or "environmental health").ti,ab,kw,kf.	110722

9	6 or 7 or 8	167420

10	5 and 9	2409

\# Web of Science Search Strategy (v0.1)

\# Database: Web of Science Core Collection

\# Entitlements:

- WOS.IC: 1993 to 2025

- WOS.CCR: 1985 to 2025

- WOS.SCI: 1900 to 2025

- WOS.AHCI: 1975 to 2025

- WOS.BHCI: 2008 to 2025

- WOS.BSCI: 2008 to 2025

- WOS.ESCI: 2020 to 2025

- WOS.ISTP: 1990 to 2025

- WOS.SSCI: 1956 to 2025

- WOS.ISSHP: 1990 to 2025

\# Searches:

1: TS=((medical or clinical or doctor* or healthcare) Near/5 (student* or educat* or school* or universit* or learn*))	Date Run: Mon Oct 06 2025 11:50:08 GMT+0100 (British Summer Time)	Results: 341487

2: TS=("climate change" or "global warming" or "global heating" or "climate crisis" or "environmental health")	Date Run: Mon Oct 06 2025 11:50:23 GMT+0100 (British Summer Time)	Results: 563134

3: #2 AND #1	Date Run: Mon Oct 06 2025 11:50:27 GMT+0100 (British Summer Time)	Results: 969

Cinahl

XB ((medical or clinical or doctor* or healthcare) N5 (student* or educat* or school* or universit* or learn*)) OR (MH "Curriculum") OR (MH "Education, Medical") OR (MH "Students, Medical")

AND

XB ("climate change" or "global warming" or "global heating" or "climate crisis" or "environmental health") OR (MH "Climate Change+") OR (MH "Environmental Health")

Global Index Medicus

("medical education" or "medical student*" or "clinical student*") AND ("environmental health" or "climate change") – searched in the Title/Abstract/Subject fields.

Results

| Database | Results 06/10/2025 |
| --- | --- |
| Medline | 1595 |
| Embase | 2409 |
| Web of Science | 969 |
| Cinahl | 549 |
| Global Index Medicus | 23 |
| After deduplication | 3403 |
